# Participatory Visual Methods with caregivers of children with Congenital Zika Syndrome in Colombia: A case study

**DOI:** 10.12688/wellcomeopenres.17529.2

**Published:** 2022-07-11

**Authors:** Veronika Reichenberger, Tracey Smythe, Shaffa Hameed, Luisa Consuelo Rubiano Perea, Tom Shakespeare, Loveday Penn-Kekana, Hannah Kuper

**Affiliations:** 1 International Centre for Evidence in Disability, Department of Clinical Research, London School of Hygiene and Tropical Medicine, London, UK; 2 Fundación Casa GAMI, Cali, Colombia; 3 Department of Public Health and Policy, Health Policy Unit, London School of Hygiene and Tropical Medicine, London, UK

**Keywords:** Participatory Video, Digital Storytelling, Participatory Visual Methods, Congenital Zika Syndrome

## Abstract

**Background**: This study explores the acceptability and feasibility of the use of two different Participatory Visual Methods (Participatory Video and Digital Storytelling) in gathering information on the experiences and perspectives of carers of children with Congenital Zika Syndrome within Colombia.

**Methods**: Participatory Video was used to assess the impact of the Juntos parent-support intervention in the lives of carers, and Digital Storytelling was used to explore the healthcare access for these children. In-depth interviews were conducted to probe participants on their views of these methods.

**Results**: One Participatory Video was produced and four Digital Stories. Of the initial eight caregivers who took part in the Participatory Video process, four completed both the Digital Storytelling process and an in-depth interview about their experiences. The main factors shaping participants’ experiences related to the skills learned in making the videos, the feeling of collectiveness and the control over the processes.

**Conclusions**: Women with children with Congenital Zika Syndrome have reported feeling marginalised and misunderstood in daily life. This case study found that Participatory Visual Methods is acceptable and feasible. Moreover, these approaches can support groups in different aspects, such as providing a space to share their stories creatively, hear others in similar situations as them and increase the feeling of community.

## Introduction

Participatory visual methods (PVM) are approaches in which research subjects develop visual material as part of the research process. PVM have roots in anthropology, as anthropologists have often used visual mediums to communicate intangible aspects of culture within their research ^1^. PVM create data that are participant-generated and give power to the participant ^2^, building a collegiate relationship between the researcher and the participant. This means that by working together, participants and researchers bring in different skills in a process of mutual learning ^3^. Accordingly, PVM can lead to a reframing of the issues discussed and generate learning from both sides, participants, and researchers alike ^4– 6^.

These participatory methodologies sustain the concepts of Paulo Freire who stated that health and its social determinants should have a wider vision and understanding, where dialogue should be promoted ^7^. As such, an appreciation of the lay knowledge and effective social participation is needed when collecting data on needs, access and impacts ^8^. As described in the WHO Toolkit on social participation, promoting social participation is important for an equitable distribution of power ^9^, including during the research process. It is especially important among marginalised people, who can then implement their own findings based on local needs ^10^. As an example, Caroline Wang described photo novellas as a way of seeking empowerment of the participants, who understand their communities best, as they identify their own issues; participatory visual methods such as photo novellas are aimed at individual change as well as improvement of the quality of life of the community ^11^. These participatory research methods complement Paulo Freire’s concept of ‘ *concientização*’ (“conscientization”), which means that the more people understand the issues within their community, the more they are in a position of power to create change ^7^. Participatory approaches are therefore important research tools; however, they require additional expertise, planning and resources. Consequently, they are often not used, particularly in low-resource settings or in relation to marginalised groups such as people with disabilities ^12^.

Two different PVM were used in this study, digital storytelling (DST) and participatory video (PV). Both processes involved a stage of reflection through group dialogue, helping to build on the stories of caregivers through sharing and listening ^13, 14^. Participants then communicate these stories through videos. DST is a process where participants create narratives telling their own stories, using a compilation of still images, videos, audio, text, and music ^10^. PV, on the other hand, involves a collaboration between participants to create group-based videos, where participants are involved in different way in crafting the video ^15^. A scope of the literature found that participatory visual approaches are most frequently used to explore the views and experiences of groups and have less commonly been used to evaluate interventions. In this paper we consider the acceptability, feasibility, and potential added value of the use of both methods by caregivers of children with congenital Zika syndrome (CZS) in Colombia.

The Zika epidemic struck in 2015, resulting in thousands of children born with microcephaly and other manifestations of CZS in Brazil and other countries in Latin America ^16, 17^. These children experience a range of health conditions, that include physical, sensory, and cognitive impairment. Consequently, they have high health care needs, which must be met from a broad range of providers, such as physiotherapy, speech and language therapists, audiologists, and occupational therapists ^18^. In addition, families are crucial to supporting the complex care needs, and they may experience strain, emotional pressure, and time constraints ^19, 20^. Colombia reported 11,944 Zika cases among pregnant women by April 2016 and was the second-most affected country after Brazil ^21^.

Health responses initially focussed on clinical needs of children with CZS; there was also a need to support caregivers in improving their skills to care for their child, as well as to provide psychosocial support to caregivers ^22^. To fill that needs gap, the Juntos programme was developed. Juntos is a participatory group programme designed for caregivers of children with CZS based on “Getting To Know Cerebral Palsy” ^23, 24^. The participatory programme targets caregivers in a support group setting and has been found to offer many important benefits, including improved understanding, confidence and self-esteem, that result in improved care for the child ^25^. Juntos was developed and piloted in Rio de Janeiro and Salvador in 2017. It was then adapted and pilot-tested in the Colombian context in 2019. To pilot Juntos for use in Colombia, data were collected from caregivers on lived experience and feasibility of implementation of Juntos, through pre-post intervention questionnaires, in-depth interviews ^26^ and PVM. The feasibility of the intervention is reported elsewhere ^26^; this paper reports on the use of PVM while evaluating the intervention.

The aim of this study was to explore the acceptability and feasibility of the use of two different PVM in gathering information on the experiences and perspectives of carers of children with CZS. PV was used to assess the impact of the Juntos parent-support intervention in the lives of carers, and DST was used to explore the healthcare access for these children. In-depth interviews were conducted to probe participants on their views of these methods.

## Methods

Data for this paper are drawn from participant and researcher experiences of the PV and the DST processes using grounded theory. These were captured through observations and semi-structured in-depth interviews with participants.

### Participatory video

The PV process was conducted in Cali, Colombia in September 2019. The aim of the PV was to explore the impact of the Juntos programme on caregivers’ lives. A group of 11 Colombian caregivers of children with CZS who had taken part in the Juntos programme were approached to take part. These 11 caregivers had formed a group on WhatsApp and were contacted by one of the Juntos facilitators present in the WhatsApp group about the participatory video. Of the 11 caregivers contacted, eight agreed to participate in the process and three refused to participate due to lack of availability. Following the InsightShare ^27^ methodology from which the facilitator did her training, a one-day workshop was led by the first author (VR). It was held at a local NGO office where the eight caregivers attended with their children. Participants consisted of six mothers, one grandmother and one sister of children with CZS.

The PV process involved a story circle, where the participants each shared their experience of the Juntos programme and how it impacted their lives. On the same day, they were taught how to film by an experienced filmmaker and facilitator (VR). With one camera available, each caregiver took turns filming another, singing to their child. They learned how to start and stop the camera, zoom, and check the sound. They then watched each practice video to understand and identify what they would want to re-create visually for the final video. Participants jointly decided through storyboarding what would be discussed in the final video, and what footage would be shot. A collective decision was made by the participant group that two caregivers would talk while others would either film or show visually what was being said in front of the camera (e.g., how to feed their child, how to play, how to make specific props learned through Juntos). All caregivers contributed to this process. Editing was then discussed, and the facilitator (VR) edited the film according to the suggestions. The final version of the film was agreed through dialogues among the facilitator and the caregivers involved, who explained what music, texts, and effects they wanted in the film. The final version was uploaded and shared with caregivers to use as they wish. The video is available in the *Extended data* ^28^.

### Digital storytelling

The DST project was conducted online in September 2020, following the Story Center ^29^ methodology at which the facilitator did her training. Of the eight mothers who took part in the PV process, six went on to participate in an initial online story circle which was undertaken through the Zoom platform, led by the first author (VR). The digital stories explored the experience of healthcare access for children with disabilities in Colombia, including reported facilitators and barriers.

During the story circle, participants shared their child’s story on healthcare access and identified a specific story that they would like to make a video about. They were then shown examples of digital stories, so they could explore how they could portray their stories visually. One week later, another session took place online where caregivers read their story and gave feedback to each other. From there, caregivers collected photos and videos to portray their story, which were sent to the facilitator (VR). Editing was completed by the facilitator, who closely followed what the participant suggested. The videos were then shared among the caregivers. Of the initial six who took part in the story circle, four completed the digital stories and three have available videos to share.

The videos are available in the *Extended data* ^28^.

### Semi-structured in-depth interviews and researcher observation notes

In total, four in-depth semi-structured interviews were conducted with participants who took part in both the digital stories and PV. Questions were asked about both methods: what participants thought of the process; how they experienced the processes; and their thoughts on the outcome. Questions included topics such as empowerment and extent of ability to communicate their message. The interviews were all conducted in Spanish by an experienced qualitative researcher (VR) via Zoom and lasted approximately 45 minutes to one hour each. All interviews were transcribed verbatim and kept in Spanish for analysis to prevent losses in translation. Transcripts were returned to participants to verify that they agreed with the content before analysis. The lead researcher’s (VR) observation notes were also included in the analysis, which included details such as the involvement of the participants, the different reactions and conversations held around the process. All transcripts and observation notes were coded manually. Coding was iterative: central themes were identified as they emerged, refined, and expanded through the coding process ^30^.

One female researcher (VR) was involved in all three phases of the study. She has previous training in both PV and DST, as well as qualitative research. She is a research assistant and PhD candidate at the London School of Hygiene and Tropical Medicine. She is of Brazilian descent and speaks Spanish fluently. She is not disabled herself and is not the carer of a child with disabilities.

### Ethics

Prior to commencing the interviews, participants were provided full information about the study, any queries answered, and written ethical consent obtained. An additional oral ethical consent was obtained at the end of both PV and DST, where participants informed the researchers whether they consented to the videos being shared/used.

Full ethical review and approval was granted by the LSHTM Ethics Committee and the Ethics approval for the study was granted by the London School of Hygiene & Tropical Medicine (LSHTM) (No 15986 /RR/ 11098) and Comité de Etica e Investigacion Asistencia Cientifica de Alta Complejidad (CEIACAC) Bogota (No CEI-022-19).

## Results

One PV and four digital stories were produced. The focus of the PV was an evaluation of Juntos, while the focus of the DST process was healthcare access. Of the initial eight caregivers who took part in the PV process, four completed both the DST process and an in-depth interview about their experiences. The other four participants did not take part in the DST and in-depth interview because of lack of time and no access to technological devices. Both participatory visual methods were acceptable and feasible, with key benefits identified in terms of the skills used to make the videos, the feeling of collectiveness and the control over the process.

### Learning and discovering new skills

None of the research participants had used a camera recorder before, and all reported using a camcorder as a new experience. All of them, but one participant, had smartphones and had previously experienced taking pictures and making videos on their phones. It was observed by the facilitator that some of the caregivers in the PV workshop were initially resistant to trying to use the camera recorder. However, once they did, and watched their videos again, the delight of watching what they filmed was evident through their facial expression, laughter, and conversations about the videos. One of the caregivers, the grandmother of one of the children (61+ years old, housewife), had little experience using any sort of technological device. She showed a particular resistance in the beginning, but also the most pride in using the camera. Other caregivers photographed her as she filmed holding her granddaughter in her arms. Two caregivers were given the responsibility of filming the PV, and when asked further questions on cinematographic techniques such as angles and ways of filming, she showed an interest in video creation.

“I learnt a lot, not only how to film but also just exploring my story in a different way, more creatively. Having to think about what I wanted to say and how I can show it in a video.” Participant 3 (30–40 years old, housewife)

Specifically, from the DST process, one participant mentioned realising she both enjoys and has a skill for writing narratives. She continued saying she had plans for doing something in the future to support parents with children with disabilities, but had not known what that would be exactly. She felt more in control of what steps to take next and what that journey might be: “After writing the narrative, I realised that I would love to write a book about my son and our story.” Participant 1 (26–30 years old, housewife)

### The collective and building community

The caregivers who took part in the PVM already knew each other from the 10-week Juntos programme. The structure of both PV and DST meant that participants reflected on common themes and built on top of each other’s stories, while having common outcomes to work towards (a digital story each or a PV). Particularly for the PV process, participants were given the space to tell one or two caregivers’ stories through a film made collectively, but they decided to change that format to something that suited them better. Through storyboarding, they derived aspects that were common to most caregivers to be portrayed in the video and made sure to include different representations (a mother feeding her daughter orally, and a mother holding her child’s gastrostomy tube). The facilitator could observe the enthusiasm of portraying their stories as one united group. It also provided a form of empowerment. For example, mothers who might otherwise shy away, wanted to be in front of the camera, mentioning they would be representing not only themselves but all mothers who may need to feed their children with a gastrostomy tube.

During the editing process, it was clear how the feeling of collectiveness and community came through. In PV, the facilitator was asked to add a slow-motion effect to one footage of all the participants walking, as they wanted to portray the strength they have in caring for their children; just like heroes are shown in films. In the digital stories, after watching each other’s videos, the participants wanted certain sections to be re-edited, so they looked more like the others, searching for similarities and unity. There was a feeling of empowerment in the collective.

“You don’t just feel like one mother, but you are *the* mothers of Cali.” Participant 2 (30–40 years old, housewife)

Adding to this feeling of community, the processes provided a space for caregivers to learn from each other and that way, change their self-perception and build strength. Their stories were heard and watched, and they could also hear and watch what others shared and made videos about.

“Participatory video is a way of expressing ideas collectively. I liked it, it was great to learn from other people’s stories and understand what is important for each one. It also supported me in understanding the journey I’m going through.” Participant 3 (30–40 years old, housewife)
“In digital storytelling, it was nice to tell and hear other people’s stories, we get to know each other better. I didn’t know how [one mother] was so resilient and what she went through in the hospital when her daughter got sick!” Participant 1 (26–30 years old, housewife)
“I enjoyed watching the different digital stories and learn more in depth what [the caregivers] went through. I thought I knew so much about the other mothers, but I realise there’s so many stories that haven’t been told. We all have so much to say.” Participant 3 (30–40 years old, housewife

Through hearing these stories, participants felt they were not alone and learned ways to overcome similar situations they may face in their day-to-day life.

### Control over the process

All caregivers reported feeling appreciated by having their ideas not only heard but implemented into their videos. They also expressed the importance of having a unique role within the different processes. Every caregiver was able to frame their stories and participate in the way they preferred.

“I’m timid, but I was able to contribute to the participatory video through sitting with [my child] and feeding her while somebody filmed us. I didn’t need to talk in front of the camera. I really liked that; I was able to contribute in my own way.” Participant 2 (30–40 years old, housewife)
“I like the way we each could express ourselves in different ways, so some mothers talked, the others were filmed feeding their children or playing. So, there was no need for everyone to do one thing, they could pick and choose within the process what they preferred.” Participant 3 (30–40 years old, housewife)

Participants were given a chance to personalise their involvement, and all caregivers were involved in storyboarding, with two leading the writing. Unlike traditional interviews, where you are expected to respond to questions posed, in a PV or DST process, you can contribute equally in other ways. Once the story was validated, four caregivers filmed the narrative (two spoke and two filmed), while the others organised the props and scenarios needed to represent the story visually. In the DST process, participants saw many examples of digital stories to be able to get ideas to create their own, and had it re-edited by the facilitator until they were fully satisfied with the outcome.

Participants reflected on the role of the facilitator, and how explaining the process as clearly and transparently as possible was key to feeling like they were truly in charge of the outcome of the videos. Fieldnotes by the facilitator reflected on the need to let go of expectations and rules attached to the different steps. For it to be a truer participatory process, the facilitator let the participants shape the process to make it theirs, while still needing to stick to the research question. The facilitator kept the “ladder of participation” in mind, where the lowest level is non-participation, the next level is partnership and the highest is effective participation ^31^. With every request to adjust the process, the facilitator felt she reached a truer level or partnership between herself and the caregivers. To have reached a higher level of participation, closer to effective participation, some things should have been decided together prior to starting the PVM, such as which PVM to use and what research question would be asked.

“I felt in control, and I could watch back and give instructions to [the facilitator]. Especially seeing other examples meant I could say what I wanted in what part of the video, and what music I wanted. After watching [one mother’s] video, there was something I liked and [the facilitator] went back and changed it for me.” Participant 1 (26–30 years old, housewife)
“[The facilitator] was there but I didn’t feel like [she] was imposing anything, we decided what we wanted and [she] helped us build it. The only thing I might have preferred was to talk about my pregnancy in the digital stories instead of health care.” Participant 4 (30–40 years old, housewife)

When comparing both methods, PV provided participants with more autonomy and individualised contribution. Within the process of DST, all participants needed to record an audio and pick visuals for the video, meaning there was less space for adjustment. While in PV, no one was obliged to do any of the steps. If caregivers did not want to share their story, they did not need to, and, as mentioned previously, they could contribute in the way they wanted. 

## Discussion

Both PVM approaches were found to be acceptable and feasible to implement with a group of caregivers of children with CZS in Colombia. Moreover, through the two PVM used, these women who have reported feeling marginalised and alone in many aspects of life ^32^, were able to find a space within these processes where they were heard and appreciated for being the experts in talking about their own lives ^33^. PVM appeared to have the capacity in this case to create what Freire calls “conscientization”, providing participants with an active role in understanding and reflecting critically on their community and life. This can lead to tackling oppression, through empowering people to question their condition and encouraging dialogue ^7^. The importance of using participatory methods in research is well recognised, including with respect to issues around disability. However, practical constraints, such as lack of tools, skills, budget, time and planning often constrain the use of participatory approaches, particularly in low- and middle-income countries (LMICs) ^12^. This case study shows that PVM are feasible and acceptable to use in this group of caregivers of children with disabilities in Colombia, even though they had very limited videography experience. The participants were not people with disabilities themselves, so that adjustments in methods were not needed. An important factor supporting feasibility was that the process was facilitated by a trained videographer who was also a qualitative researcher and Spanish speaker. Participation was particularly observed during data collection, whereas the analysis (video editing) was led by the videographer, but with high levels of input from the participants.

PVM provide space for dialogue in many forms, such as dialoguing in a group and in the video itself. In this project, importance was given not only to expressing oneself but also being heard and hearing others. Low and colleagues talk about the act of being listened to as being one of the main transformational effects for individuals taking part in a process such as DST ^34^. Many facilitators have identified hearing and being heard as one of the main empowerment aspects of these methods as well; having a safe and supportive space to share your story, regardless of who will watch the final films ^35, 36^. This was also observed by the facilitator in this study.

Recent approaches define empowerment as a change in self-perception and increased control over different areas of one’s life ^37, 38^. This definition of empowerment encapsulates what was reported from this study, where participants’ awareness of themselves and their own community grew through the process, as they learned from one another and picked up new skills that may lead to further outcomes, such as the mother who now wants to write a book for other caregivers of children with disabilities. This form of empowerment in participants has also been seen in previous studies involving PVM ^39– 42^. Within this case study, DST and PV have the common goal of empowering individuals and their communities, with an emphasis on the process over the product ^43, 44^. In their interviews, participants clearly focused on the process of dialoguing and making the videos, more than the final film itself. The act of giving participants a tool that they can themselves use to create, instead of creating something for them, is very powerful. This has been discussed by Yoly Gutierrez when explaining the experience of giving a camera to members of the Kayapó Indigenous tribe ^45^. Tribesmen described the camera as their “weapon”, as it has the power to strengthen them through providing a tool to voice their experiences as well as giving them a medium to communicate with people outside their community ^45^. Mtuy *et al.* also discussed this in a Photovoice study conducted with Maasai women, where women felt empowered as they were put in a role of “educators, agents of change and a source of valued information.” ^46^. In one of her papers, Caroline Wang talks about an empowerment project that took place in Peru, where women used pencils for the first time. That helped build confidence in women who had never used pencils before ^11^. Women are recognised and appreciated for being experts in their own lives and communities.

Regarding the role of the facilitator within a participatory process, the facilitator must have the capacity to let go and form a collegiate relationship with the participants ^3^. The participants should be able to contribute to shaping certain aspects of the study, with the facilitator there to support that creation ^33^. Facilitators should reflect on the nature of oppression and actively tackle it in all stages of participatory research ^7^. In this case study, it was decided at the start that the facilitator would edit videos, which was agreed on by participants who had limited time to edit due to many factors, but that the facilitator would work as a ‘tool’ to create the video as the participants wanted. It is important to have an intimate and non-hierarchical relationship so that participants can be involved and accept the goals of the research project ^47^. For this, dialogue and transparency are important in all stages of the study.

The two main limitations of this study that might have restrained the full potential for understanding the experience of PVM, were time and number of participants. Both PV and DST processes for this study were completed over a short period of time due to time restraints. While some PV projects may take months, both PV and DST for this study took 2.5–3 weeks to complete. Additionally, only four participants were involved in both methods forming the basis for this case study. Therefore, the results cannot be extrapolated to a larger group. If more participants were interviewed and involved, more themes may have emerged and would have allowed inter-group comparisons (e.g., differences by gender, age, support from family, etc). Additionally, the structure of the methods themselves may have inhibited more effective form of empowerment or impact. This has been seen in other PVM such as Photovoice projects ^48^. One example was the research question of the DST process; one mother made it very clear that she would have preferred talking about her experience during pregnancy rather than focusing the videos on the initial research question, which was on health care access for their children. The other mothers agreed. On a Photovoice project undertaken by Caroline Wang, she points out that the method itself might have “stopped short of engaging participants in conceptualising and participating in action steps needed to address their needs.” ^40^

## Conclusions

Women with children with CZS have reported feeling marginalised and misunderstood in daily life. This case study shows that PVM are acceptable and feasible, and moreover supported this group through learning and discovering new skills, building the feeling of collectiveness, and providing a space to share their stories creatively.

## Data availability

### Underlying data

As participants are a close group of caregivers in Cali, the content of the interviews includes names of other caregivers and their children, information, addresses and personal stories. Some of the information mentioned are of participants who were not interviewed for this paper; therefore, it would not be ethical to share this information as they have not consented to it.

### Extended data

LSHTM Data Compass: Participatory Visual Methods with caregivers of children with Congenital Zika Syndrome in Colombia: A case study, https://doi.org/10.17037/DATA.00002745 ^28^

This project contains the following extended data:

- La discapacidad en medio de una sala de urgencias
- Falta_de_tacto_de_algunos_profesionales
- Gabriela_venciendo_el_Zika
- PV_Colombia_subs

'PV_Colombia_subs' is a participatory video produced by caregivers of children with Congenital Zika Syndrome in Cali, Colombia. Through the workshop and video production, caregivers explored the impact of the 'Juntos' program on their lives. All participants featured in the video consented to their images and children's images being shared.

'La discapacidad en medio de una sala de urgencias' 'Falta_de_tacto_de_algunos_profesionales' and 'Gabriela_venciendo_el_Zika' are three digital stories where caregivers of children with Congenital Zika Syndrome explore one story of their child's healthcare access journey. The videos were created within the Digital Storytelling structure. All participants consented to their video being shared.

Data are available under the terms of the Creative Commons Attribution-NonCommercial-NoDerivs 3.0 Unported license (CC BY-NC-ND 3.0).
