# Supplementary material for: Participatory Visual Methods with caregivers of children with Congenital Zika Syndrome in Colombia: A case study
Source: Wellcome Open Res. 2022 Jul 11;7:107. Originally published 2022 Mar 23. [Version 2] doi: 10.12688/wellcomeopenres.17529.2 (PMC10620479; doi:10.12688/wellcomeopenres.17529.2)
Supplement: Peer review history

# Reviewer report: Reviewer response for version 2

**DOI:** 10.21956/wellcomeopenres.19976.r67827

**Published:** 2023-11-01

**Authors:** Andrea Mantsios

**Affiliations:** 1 Public Health Innovation & Action, New York, USA

Thank you for the opportunity to review this manuscript.

 This paper describes the acceptability and feasibility of two different participatory visual methods to capture experiences and perspecttives of caregivers of children with Congenital Zika Syndrome (CZS) in Colombia. It is a well-written and well-organized manuscript detailing the use of less commonly used qualitative methods (e.g., participatory video and digital storytelling) along with in-depth interviews to understand participant experiences with these methods.  

 One of my suggestions for the authors echos comments made by the previous reviewers and has to do with better understanding the group of women who ended up participating as well as those who did not, chose to decline. Given this is a case study with a small sample, I would have loved if the authors described the women who participated in greater detail to give us more of a sense of their lives and circumstances to better understand who they are more broadly as we think about the experiences they had engaging with these methods. We, of course, learn about the women highlighted in the products of this work shared in the Extended Data, but it would be helpful to get a sense of the women who participated in the study in the article to further contextualize the experiences they had. It was mentioned that the women who did not participate in the interviews did not have access to technological devices but it was also mentioned that all but 1 of the 8 women in the PVM portion of the study had smartphones which I think would have facilitated conducting the IDIs via zoom. Similarly, I’m wondering if efforts were made to conduct IDIs locally so that tech access was not a barrier to participation. In my own work, I am particularly conscious of the equity issues that arise in conducting qualitative research virtually and who may be excluded from having their voices heard and represented in the findings because of that barrier. Just a note to consider and reflect on in the limitations of this work.

 Overall, I think this paper is a valuable contribution to the literature and appreciate the opportunity to provide my thoughts.

Is the work clearly and accurately presented and does it cite the current literature?

Yes

If applicable, is the statistical analysis and its interpretation appropriate?

Not applicable

Are all the source data underlying the results available to ensure full reproducibility?

Yes

Is the study design appropriate and is the work technically sound?

Yes

Are the conclusions drawn adequately supported by the results?

Yes

Are sufficient details of methods and analysis provided to allow replication by others?

Yes

Reviewer Expertise:

Qualitative research, caregivers of children with CZS in Brazil, HIV

I confirm that I have read this submission and believe that I have an appropriate level of expertise to confirm that it is of an acceptable scientific standard.

# Reviewer report: Reviewer response for version 2

**DOI:** 10.21956/wellcomeopenres.19976.r51477

**Published:** 2022-08-26

**Authors:** Debora Diniz

**Affiliations:** 1 The Center for Latin American and Caribbean Studies, Brown University, Providence, RI, USA

Thanks for submitting the article to my second reading. 

 The authors did a great work addressing the comments and making clearer some of the previous questions, particularly regarding the methods and the participants description. 

 The article should be approved.

Is the work clearly and accurately presented and does it cite the current literature?

Yes

If applicable, is the statistical analysis and its interpretation appropriate?

Not applicable

Are all the source data underlying the results available to ensure full reproducibility?

Yes

Is the study design appropriate and is the work technically sound?

Partly

Are the conclusions drawn adequately supported by the results?

Partly

Are sufficient details of methods and analysis provided to allow replication by others?

Yes

Reviewer Expertise:

My area of research is also Zika, women and visual methods

I confirm that I have read this submission and believe that I have an appropriate level of expertise to confirm that it is of an acceptable scientific standard.

# Reviewer report: Reviewer response for version 1

**DOI:** 10.21956/wellcomeopenres.19381.r50828

**Published:** 2022-06-20

**Authors:** Ignacio Zarante

**Affiliations:** 1 Human Genetics Institute, Pontificia Universidad Javeriana, Bogota, Colombia

This is an article where participatory visual methods are used in families of patients with sequelae of prenatal Zika virus infection in the city of Cali, Colombia. They search for 11 families, but in the end they only manage to complete the qualitative research in 4 of them. Families should develop a digital storytelling or participatory video. Each caregiver and family was taught how to operate the camera and instructed to develop a script with the help of the researchers. These scripts were developed together and shared with the other families the experience they were developing. At the end, the information was analyzed using semi-structured in-depth interviews.

 The results are remarkably interesting since the whole process generated participatory work within the family and contact with the others. It is evident that this strategy has a great feasibility to empower families with members who are disabled. The limitation of the methods was in a small sample that also ended with a small number of participants. The conclusions are interesting, but it is not so easy to extrapolate the results to larger groups. The authors do not give much information on the reasons why some of those involved did not participate and there may be explanations for the usefulness of these strategies.

Is the work clearly and accurately presented and does it cite the current literature?

Yes

If applicable, is the statistical analysis and its interpretation appropriate?

Not applicable

Are all the source data underlying the results available to ensure full reproducibility?

Yes

Is the study design appropriate and is the work technically sound?

Partly

Are the conclusions drawn adequately supported by the results?

Partly

Are sufficient details of methods and analysis provided to allow replication by others?

Yes

Reviewer Expertise:

Birth defects, medical genetics, public health

I confirm that I have read this submission and believe that I have an appropriate level of expertise to confirm that it is of an acceptable scientific standard, however I have significant reservations, as outlined above.

# Response

**Published:** 2022-06-29

**Authors:** Veronika Reichenberger

**Affiliations:** London School of Hygiene & Tropical Medicine, UK

Dear Ignacio Zarante,

 Thank you for your comments and review. We are honoured to get your important feedback on our paper.

 We are also very pleased to read you found the results interesting.

 With regards to not being able to extrapolate to a larger group, we have added a sentence clarifying this in the last paragraph of the results, which now states:

 “Additionally, only four participants were involved in both methods forming the basis for this case study. Therefore, the results cannot be extrapolated to a larger group.”

 We are please to inform that we have also added more information on the reasons why we did not have more participants involved. A new sentence found in the first paragraph of the results section, now states:

 "The other four participants did not take part in the DST and in-depth interview because of lack of time and no access to technological devices."

 We hope to have provided appropriate clarification and are available for further exchanges and modifications where needed.

# Reviewer report: Reviewer response for version 1

**DOI:** 10.21956/wellcomeopenres.19381.r49405

**Published:** 2022-04-13

**Authors:** Debora Diniz

**Affiliations:** 1 The Center for Latin American and Caribbean Studies, Brown University, Providence, RI, USA

The research article describes the methodological processes involved in using visual research tools with women affected by the Zika epidemic in Colombia. It's a descriptive study about how the visual tools facilitated gathering information about the research question. The group of women involved in this research  initiative was small and some of them decided to step down during the process, which was not clarified by the authors. I appreciate the opportunity to read the manuscript in advance and I hope that my comments will help the authors to further develop such an important initiative:

1. Power dynamics, Empowerment

 I would not describe the visual tools as mechanisms of "giving power" to participants. It's more about sharing than giving, and the replacement of verbs here is not just rhetorical: it is also deeply conceptual. It is about how the researchers understand their roles and purpose in using visual tools in the context of a research interaction. Why am I mentioning this point? The study presents two purposes for using visual tools in social research: as a way of engaging participants and as a tool for "concientizacao" or "empowerment" (as presented in the manuscript, these two concepts are not interchangeable; but the content of each one is not that clear). The first purpose is clearly stated in the descriptive narrative about the processes involved in working with different visual methodologies. The second purpose is not that straightforward, however, and I wonder if the authors should consider eliminating it. There is no evidence in the study that the visual methods empowered women: the example of one of the participants referring to "writ[ing] a book about her son" is more a regional way of expressing the idea that "I'm going to tell my story" than the literal intention to initiate a new project. I do not see the meetings for visual literacy as reflecting the concept of engaged pedagogy conceptualized by Paulo Freire or bell hooks. Finally, in my opinion, the concept of "hearing" and "being heard" by others is a key emic concept yet it was not explored by the authors as a key element of using visual methodologies.

2. Caregivers, women, disability

 The study was conducted among women. Yet gender is absolutely ignored by the manuscript. The study refers to them as "caregivers" or "mothers"  or “grandmother”. We have no other information about them, just that their visual literacy was low - an observation that I question considering the levels of digital inclusion in Colombia via the use of smartphones. We need to know more about who these women are, beyond their social roles as caregivers of children affected by the Zika congenital syndrome. In a brief reflective sentence about the positionality of the facilitator, s/he mentions that s/he is not disabled nor a carer of a child with disability. But why only mention disability here if the work was among women? Why not also consider race, class, education, gender, nationality, as crucial factors to understand the research framing  and the power dynamics? 

3. Minor comments

 I would encourage the authors to confirm if there is any updated data about Zika cases among pregnant women in Colombia. The study mentions April 2016 as the reference. I would also consider reorganizing the arguments, in case the authors prefer to maintain the argument of empowerment/conscientizacao. If they keep that argument, my recommendation would be to incorporate it as part of the analysis of the results and not as an assumption in the Introduction.

 I deeply admire the study and the ethical commitment that led the authors to work with visual methods, particularly recognizing how rare and demanding it is. I'm more than happy and available to reconsider my comments if the authors do not consider them appropriate for what they have in mind.

Is the work clearly and accurately presented and does it cite the current literature?

Yes

If applicable, is the statistical analysis and its interpretation appropriate?

Not applicable

Are all the source data underlying the results available to ensure full reproducibility?

Yes

Is the study design appropriate and is the work technically sound?

Partly

Are the conclusions drawn adequately supported by the results?

Partly

Are sufficient details of methods and analysis provided to allow replication by others?

Yes

Reviewer Expertise:

My area of research is also Zika, women and visual methods

I confirm that I have read this submission and believe that I have an appropriate level of expertise to confirm that it is of an acceptable scientific standard, however I have significant reservations, as outlined above.

# Response

**Published:** 2022-06-29

**Authors:** Veronika Reichenberger

**Affiliations:** London School of Hygiene & Tropical Medicine, UK

Dear Debora Diniz,

 Thank you very much for your review and request for clarification. It’s an honour to receive a review from such a prestigious academic as yourself.

1. Power dynamics, Empowerment

 We would like to clarify our reasoning behind mentioning empowerment in the introduction. We were pleased to include background in the introduction to assist the reader in understanding certain concepts such as empowerment, which is a recognized concept within PV (as referenced by Caroline Wang in her extensive work with participatory visual methods such as photovoice).

 With regards to writing the book, we understand the space for interpretation this left. We would like to clarify that beyond mentioning wanting to write a book, the participant went into more detail, including questions regarding publishing, which supported the understanding of wanting to write a book as opposed to an expression.

2. Caregivers, women, disability

 As suggested, we provided more information about who the women are after the quotes to assist the readers. We have added age group and occupation. (Example: Participant 2, 30-40 years old, housewife)

 We are pleased to highlight that the reflective sentence does include more details about the facilitator, as mentioned in the last paragraph before the ethics, in the methods section:

 “One female researcher (VR) was involved in all three phases of the study. She has previous training in both PV and DST, as well as qualitative research. She is a research assistant and PhD candidate at the London School of Hygiene and Tropical Medicine. She is of Brazilian descent and speaks Spanish fluently. She is not disabled herself and is not the carer of a child with disabilities.”

 We have also added a sentence to clarify about the participants visual literacy:

 “All of them, but one participant, have smartphones and have previously experienced taking pictures and making videos on their phones.”

 We also added “cinematographic techniques” to support the understanding that the new skills were specific to these techniques (lighting, angles, using a camcorder) as opposed to making a video itself, which all but one had already done.

 Thank you for these important comments and requests.

3. Minor comments

 We would like to clarify the reasoning behind the 2016 status. The zika epidemic went from 2015 to 2016, which is when Congenital Zika Syndrome was identified. This is hence the focus of our study and believe the statistics from 2016 provide better understanding on the background of the study than recent statistics.

 We are honoured to hear you admire the study and recognize how rare and demanding it can be.

 We hope to have clarified your comments and are available for further exchanges and clarifications where needed.
